# Inactivated vaccine injection and immunoglobulin G levels related to severe coronavirus disease 2019 (Delta) pneumonia in Xi’an, China: A single-centered, retrospective, observational study

**DOI:** 10.3389/fcimb.2022.933100

**Published:** 2022-08-23

**Authors:** Han Yang, Jinbao Ma, Aifang Li, Jing Lei, Fenqing Shang, Yue Cheng, Bei Han, Hongbo Li, Yuee Chen, Yuanli Yang, You Xu

**Affiliations:** ^1^ Medical Transformation Center of Xi’an Chest Hospital, Xi’an, China; ^2^ Department of Tuberculosis, Xi’an Chest Hospital, Xi’an, China; ^3^ Department of Laboratory Medicine, Xi’an Chest Hospital, Xi’an, China; ^4^ School of Public Health, Xi’an Jiaotong University, Xi’an, China; ^5^ Institute of Respiratory Disease Research, Xi'an Chest Hospital, Xi'an, China

**Keywords:** COVID-19, vaccine, booster shot, Immunoglobulin G, severity

## Abstract

The World Health Organization declared a public health emergency of international concern in January 2020. The Delta variant became the main epidemic strain on 11 May 2021. Vaccines were proven highly effective in controlling hospitalization and deaths associated with severe acute respiratory syndrome coronavirus 2 infections. Real data on vaccine efficacy against B.1.617.2 infection in the Chinese population were currently limited. This study aimed to evaluate the protective effect of inactivated vaccine injection and immunoglobulin (Ig) G levels in coronavirus disease 2019 (COVID-19) severity. This retrospective study included patients with COVID-19 in Xi’an Chest Hospital from December 2021 to January 2022. The protective effect of inactivated vaccine injection and IgG levels on COVID-19 severity was analyzed using multiple logistic regressions. A total of 580 patients were included in the study, of whom 158 (27.24%) were mild, 412 (71.03%) were moderate, 5 (0.9%) were severe, and 5 (0.86%) were critical. Severe case (including severe and critical) rates were 1.72% (10/580). Compared with the unvaccinated group, the vac+IgG− group had a 0.21 (0.02–2.05)-fold risk of suffering from severe cases, and the vac+IgG+ group had a 0.05 (0–0.63)-fold risk of suffering from severe cases. Of the 10 severe cases, 8 were older than 60 years, 8 were men, 8 had underlying diseases, 6 were in the unvaccinated group, and 2 were in the vac+IgG− group. Vaccination and sufficient IgG antibody production can protect patients with COVID-19 from severe cases. Booster vaccine injection can produce a stronger immune response and protection.

## Introduction

Coronavirus disease 2019 (COVID-19) is a severe respiratory syndrome in humans caused by severe acute respiratory syndrome coronavirus 2 (SARS-CoV-2), a single, positive-strand ribonucleic acid (RNA) virus (Holmes, 2003). The World Health Organization (WHO) declared a public health emergency of international concern in January 2020 (Li et al., 2020; Wrapp et al., 2020). SARS-CoV-2 had become more transmissible and virulent in evolution and mutation. The WHO Technical Advisory Group on Viral Evolution listed the Delta variant as a variant of interest (VOI) on 4 April 2021, which was further classified as a variant of concern (VOC) on 11 May 2021 (Tao et al., 2021; Tian et al., 2021; World Health Organization, 2021b).

Vaccines have been proven highly effective in controlling COVID-19-related hospitalization and deaths, but the emergence of viral variants with novel antigenic profiles threatens to diminish their efficacy. The following three types of COVID-19 vaccines are widely used: messenger RNA (mRNA)-based, viral vector (non-replicating) vaccine, and inactivated vaccines. In terms of the efficacy against overall infection by the Delta variant, RNA-based vaccines ranked first, followed by the viral vector (non-replicating) and inactivated vaccines, which is consistent with the results against the original COVID-19 strain (Cai et al., 2021; Davis et al., 2021; Lopez Bernal et al., 2021). Vaccination cannot completely prevent infection by the Delta variant; however, protection against moderate and severe infection remains satisfactory, and all vaccines exhibit efficacy of >70%. Vaccination can prevent death and greatly reduce the proportion of severe cases compared to those unvaccinated. However, the efficacy of all vaccines was decreased against the Delta variant. The level of neutralizing antibodies is an important factor in determining the effectiveness of vaccines.

In the context of China’s effective viral epidemic control, the Delta variant only spread on a small scale in various places in 2021, with a small number of infected people, and the epidemic due to the Delta variant ended by the end of 2021 (Wang et al., 2021). Then, Omicron became the main epidemic strain in March 2022. Clinical studies on the prevalence of Delta variant strains in China were limited, mainly due to the small number of infected people and the short epidemic cycle. The epidemic scale of Delta B.1.617.2 variant (hea.china.com, 2021) in Xi’an, Shaanxi Province, was approximately 2,000 people, which was a virus epidemic under the background of a high vaccination rate (inactivated vaccine). As of 18:00 on September 2, Xi’an had a total of 22.41 million doses of inoculation, 11.03 million in the first dose, 10.45 million in the second dose, and 923,000 in the third dose; according to the seventh census data of 11.10 million people, the coverage rate of the first dose of the population over 12 years old is 99.38%, and the coverage rate of the second dose is 94.14% (http://xa.bendibao.com/live/2021525/84943.shtm). The human body’s immune response to vaccines is differentiated due to the body’s immune status, vaccine doses, vaccination time, and other reasons resulting in different neutralizing antibody levels, especially the specific antibody immunoglobulin (Ig) G, which is crucial in the long-term body protection (Painter et al., 2015; Zimmermann and Curtis, 2019; Trevisan et al., 2020).

Real data on vaccine efficacy (VE) against B.1.617.2 infection in the Chinese population were currently limited. This study collected patients’ electronic medical records, including population characteristics, underlying diseases, laboratory test results within 48 h of admission, chest computed tomography (CT) results, and final diagnostic classification. The level of IgG antibody production after vaccination and the protective effect on severe diseases were evaluated by classifying the vaccination status of the included patients, combined with the IgG results.

## Methods

### Study design and participants

This retrospective observational study included patients with COVID-19 infection in Xi’an Chest Hospital from December 2021 to January 2022. All patients were positive for a pharyngeal swab or nasopharyngeal swab COVID-19 nuclear acid test. Xi’an City, located in northwest China, is the capital of Shaanxi Province, with a population of 12 million. Xi’an Chest Hospital is a tuberculosis specialist hospital and has been designated a hospital for COVID-19 since December 2021. As reported, >2,000 patients were diagnosed with COIVD-19 in Xi’an from December 2021 to January 2022. Additionally, 661 cases were inpatient in Xi’an Chest Hospital. Patients under 18 years old and with missing IgG data were excluded from the study. Inactivated vaccine injection, IgG levels, and baseline data were collected, and univariate and multivariate analyses were used to assess the association of inactivated vaccine injection and IgG levels with severe COVID-19 pneumonia.

### Data collection and measurements

All data were collected from the electronic medical system of patients who are tested within the first 48 h of admission, including population characteristics (gender, age, height, and weight), comorbidities (diabetes, hypertension, heart disease, hepatitis, immune disease, lung disease, and tumor), laboratory test results (routine, liver, kidney, cardiovascular, and coagulation functions), chest radiographic findings (lung lesion distribution), and diagnostic classification. The study was approved by the Ethics Committees of Xi’an Chest Hospital in March 2022 (No. S2022-0002), which granted permission for the use of anonymized patients and exempted the informed consent.

### Laboratory tests

COVID-19 IgG antibodies were detected using the following serological tests: an automated chemiluminescent microparticle immunoassay (CMIA) test for the qualitative detection of IgG antibodies to the spike protein of COVID-19 (Maccura Co. Ltd., Cat: IM4409013, China). Serum samples were considered positive when the output index was ≥1.0 S/CO according to the manufacturer’s instructions.

### Definition

Patients were classified into mild, moderate, severe, and critical groups according to disease severity (World Health Organization, 2021a). Mild COVID-19 pneumonia was defined as patients with a positive swab COVID-19 nucleic acid test, with COVID-19-related symptoms and an absence of lung lesion. Moderate COVID-19 pneumonia was defined as patients with a positive swab COVID-19 nucleic acid test, COVID-19-related symptoms, and lung lesion. Severe COVID-19 pneumonia was defined as patients suffering from one of the following conditions: respiratory rate of >30 breaths/min, oxygen saturation of ≤93% at a rest condition, arterial oxygen partial pressure/fractional inspired oxygen ratio of ≤300 mmHg, and lung lesion increased for >50% in 24–48 h. Critical COVID-19 pneumonia was defined as patients suffering from one of the following conditions: respiratory failure requiring mechanical ventilation, under shock, and multiple organ failure.

Patients were classified into three categories according to vaccine injection and IgG level. Unvaccinated was defined as patients who did not have any COVID-19 vaccine injection. Vac+IgG− was defined as patients who have been injected with the inactivated vaccine with a negative IgG test. Vac+IgG+ was defined as patients who have been injected with the inactivated vaccine with a positive IgG test. IgG type includes unvaccinated, vac+IgG−, and vac+IgG+. Severe cases in the univariate and multivariate analyses were defined as patients who were diagnosed with severe and critical COVID-19.

### Data analysis

Data were analyzed using the Statistical Package for the Social Sciences version 26.0 (IBM Inc. 2019, New York, USA) and R. version 3.3.2 (http://www.R-project.org, The R Foundation, TUNA Team, Tsinghua University, China). *P*-values of <0.05 (two-sided) were considered statistically significant. Missing data were analyzed, and missing data of <5% were not included. Firstly, baseline characteristics were shown according to vaccine and IgG. Continuous data were measured as means with standard deviations (SDs) or medians with an interquartile range (IQR) according to the normality of distribution. Categorical variables were measured with frequency and percentages. Continuous variables were compared using the one-way ANOVA test, and the Kruskal–Wallis test was used for abnormally distributed variables. Categorical variables were compared with the chi-square tests or Fisher’s exact test. Secondly, univariate analysis was performed to select potential risk factors. Thirdly, multivariate logistical regression analysis was used to assess the relationship between IgG type and disease severity. We created four models including non-adjusted and multivariate models. Confounders included factors of clinical interest and significant variable in the univariate analysis. At last, we showed the details of 10 severity cases.

## Results

### Baseline characteristics and antibody levels for different vaccine doses

A total of 661 patients with positive COVID-19 nucleic acid tests were admitted to Xi’an Chest Hospital from December 2021 to January 2022. Baseline characteristics by age group are shown in **Table S1**. Among them, 74 were younger than 18 years, and 7 without a test for COVID-19 IgG antibodies were excluded. Hence, a total of 580 patients were included in the study. Baseline characteristics by IgG type are shown in **Table 1**. Among them, 60 patients were not vaccinated against COVID-19, 115 were vaccinated and were negative for COVID-19 IgG antibodies, and 405 were vaccinated and positive for COVID-19 IgG antibodies. The mean age was 40.0 ± 14.3 years; 302 (52.07%) were men, 278 (47.93%) were women, and 102 (17.59%) had underlying diseases. The final diagnosis revealed that 158 (27.24%) cases were mild, 412 (71.03%) were moderate, 5 (0.86%) were severe, and 5 (0.86%) were critical. Vaccination status and IgG antibody levels are shown in **Table 2**. The IgG level of booster vaccine injection was 8.90 S/CO (7.14–9.58), higher than partly, full vaccine injection, and unvaccinated. The IgG test was positive in all of the booster vaccine injection patients (50/50).

**Table 1:** Baseline characteristics of the patients.

Variables	Total, *n* = 580	Unvaccinated, *n* = 60	Vac+IgG−, *n* = 115	Vac+IgG+, *n* = 405	*p*-value
Sex					0.013
Female	278 (47.9)	31 (51.7)	41 (35.7)	206 (50.9)	
Male	302 (52.1)	29 (48.3)	74 (64.3)	199 (49.1)	
Age, years	40.0 ± 14.3	47.0 ± 17.8	41.6 ± 15.8	38.5 ± 12.8	<0.001
BMI, kg/m^2^	23.7 ± 3.6	23.5 ± 3.3	24.0 ± 3.8	23.7 ± 3.6	0.656
Injection time, days	180 (142, 189)	NA	185 (156, 193)	166 (136, 188)	<0.001
Symptoms					
Fever	278 (47.9)	37 (61.7)	48 (41.7)	193 (47.7)	0.043
Fatigue	83 (14.3)	10 (16.7)	19 (16.5)	54 (13.3)	0.593
Sore_throat	270 (46.6)	26 (43.3)	47 (40.9)	197 (48.6)	0.293
Cough	378 (65.2)	39 (65)	74 (64.3)	265 (65.4)	0.977
Expectoration	215 (37.1)	20 (33.3)	42 (36.5)	153 (37.8)	0.794
Nasal_rhinorrhea	149 (25.7)	9 (15)	28 (24.3)	112 (27.7)	0.104
Impaired_smell_taste	93 (16.0)	11 (18.3)	18 (15.7)	64 (15.8)	0.876
Headache	44 (7.6)	6 (10)	8 (7)	30 (7.4)	0.709
Diarrhea	32 (5.5)	8 (13.3)	8 (7)	16 (4)	0.012
Dyspnea	70 (12.1)	11 (18.3)	16 (13.9)	43 (10.6)	0.183
Asymptomatic	20 (3.4)	2 (3.3)	7 (6.1)	11 (2.7)	0.185
D-dimer, 0–1 μg/ml	0.4 (0.4, 0.5)	0.5 (0.4, 0.6)	0.4 (0.3, 0.5)	0.4 (0.4, 0.5)	<0.001
PT, 11.8–15.1 s	13.4 (12.3, 14.4)	13.2 (12.4, 14.5)	13.2 (12.0, 14.4)	13.5 (12.5, 14.4)	0.212
APTT, 25.4–38.4 s	32.1 (30.1, 34.0)	32.1 (30.1, 34.1)	31.9 (29.6, 33.9)	32.2 (30.2, 34.0)	0.689
INR, 0.7–1.4	1.1 (0.9, 1.1)	1.0 (1.0, 1.1)	1.0 (0.9, 1.1)	1.1 (1.0, 1.1)	0.211
IgM, <1.0 S/CO	0.4 (0.1, 4.5)	0.1 (0.0, 0.3)	0.0 (0.0, 0.1)	1.5 (0.2, 7.1)	<0.001
Albumin, 40–55 g/L	46.4 ± 3.4	45.4 ± 4.1	47.0 ± 3.1	46.4 ± 3.4	0.013
Globulin, 20–40 g/ml	25.1 ± 3.8	25.4 ± 4.4	25.0 ± 3.7	25.1 ± 3.8	0.739
Myoglobin, 0–146.9 ng/ml	29.0 (21.7, 40.5)	35.0 (24.3, 48.4)	29.6 (22.0, 41.5)	28.6 (21.3, 39.7)	0.047
CKMB, 0–4 ng/ml	0.4 (0.3, 0.7)	0.4 (0.3, 0.8)	0.5 (0.3, 0.8)	0.4 (0.3, 0.6)	0.196
WBC, 3.5–9.5 × 10^9^/L	4.8 (3.9, 5.9)	4.5 (3.7, 5.5)	5.2 (4.1, 6.6)	4.8 (3.9, 5.8)	0.002
Lymphocytes, 1.1–3.2 × 10^9^/L	1.3 (0.9, 1.7)	1.1 (0.8, 1.5)	1.3 (0.9, 1.7)	1.3 (1.0, 1.7)	0.115
Monocytes, 0.1–0.6 × 10^9^/L	0.4 (0.3, 0.5)	0.4 (0.3, 0.5)	0.5 (0.4, 0.6)	0.4 (0.3, 0.5)	<0.001
Platelets, 125–350.0 × 10^9^/L	196.0 (155.0, 236.0)	180.5 (151.5, 209.2)	205.5 (164.2, 243.5)	195.0 (154.0, 237.0)	0.056
Hemoglobin, 115–150 g/L	141.0 (127.0, 153.0)	135.0 (124.0, 147.0)	149.0 (136.0, 159.0)	140.0 (127.0, 153.0)	<0.001
CRP, 0–6 mg/L	6.5 (3.6, 16.7)	6.8 (3.5, 19.9)	5.1 (3.2, 12.7)	7.2 (3.8, 17.5)	0.022
ESR, 0–15 mm/h	21.5 (12.0, 36.0)	22.0 (16.0, 40.0)	15.0 (7.0, 26.5)	22.5 (13.0, 39.2)	<0.001
Comorbidities	102 (17.6)	19 (31.7)	26 (22.6)	57 (14.1)	0.001
Diabetes	16 (2.8)	2 (3.3)	5 (4.3)	9 (2.2)	0.34
Hypertension	55 (9.5)	12 (20)	15 (13)	28 (6.9)	0.002
Coronary_heart_disease	10 (1.7)	3 (5)	4 (3.5)	3 (0.7)	0.01
Chronic_hepatitis	14 (2.4)	2 (3.3)	1 (0.9)	11 (2.7)	0.485
Other_immune_diseases	11 (1.9)	6 (10)	1 (0.9)	4 (1)	<0.001
Lung_disease	14 (2.4)	0 (0)	4 (3.5)	10 (2.5)	0.392
Tumor	5 (0.9)	1 (1.7)	3 (2.6)	1 (0.2)	0.031
Clinical classification					<0.001
Mild	158 (27.2)	9 (15)	30 (26.1)	119 (29.4)	
Moderate	412 (71.0)	45 (75)	83 (72.2)	284 (70.1)	
Severe	5 (0.9)	4 (6.7)	0 (0)	1 (0.2)	
Critical	5 (0.9)	2 (3.3)	2 (1.7)	1 (0.2)	
Conversion time, days, median (IQR)	18.0 (15.0, 22.0)	19.0 (16.0, 23.0)	20.0 (16.0, 22.0)	18.0 (15.0, 21.0)	0.006
Chest CT					0.032
Unilateral lesion	110 (21.0)	5 (8.8)	19 (18.8)	86 (23.6)	
Bilateral lesions	413 (79.0)	52 (91.2)	82 (81.2)	279 (76.4)	

Data are median (IQR) or *n* (%).

**Table 2:** Antibody levels for different vaccine doses.

Variable	Vaccine dose				Total	*p*-value
	Unvaccinated, *n* = 60	Partial, *n* = 21	Full, *n* = 449	Booster, *n* = 50		
IgG (S/CO)	0.07 (0.01–1.82)	8.46 (1.81–9.56)	7.93 (1.16–8.87)	8.90 (7.14–9.58)	7.79 (0.79–8.9)	<0.001
IgG+ (*n*, %)	20, 33.33%	16, 76.19%	340, 75.72%	50, 100%	426, 73.45%	

### Risk factors of COVID-19 severe cases

**Figure 1:**
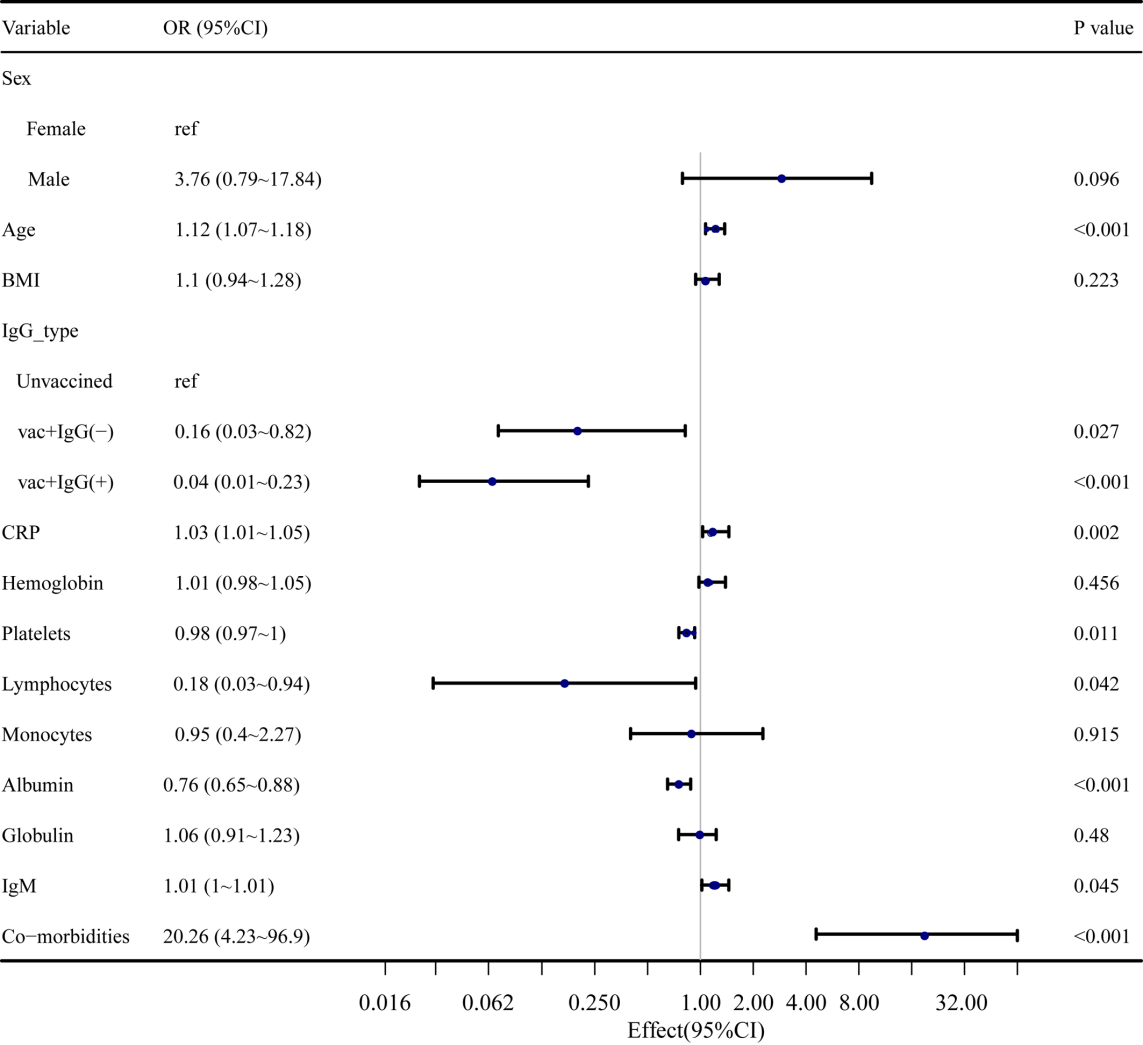
Univariate analysis of risk factors of severe cases.

According to the type of diagnosis, patients were divided into severe cases, which included 10 patients who were diagnosed as severe and critical, and non-severe cases, which included 570 cases. The results of the univariate analysis that developed into severe cases are shown in **Figure 1**. The odds ratio (OR) values of IgG type, platelet, albumin, and lymphocyte were <1, respectively, and the OR values of age, C-reactive protein (CRP), COVID-19 IgM, and comorbidities were >1, respectively. The relevant data are shown in detail in **Table S2**.

### Protective effects of inactivated vaccine injection and IgG levels on the development of severe pneumonia

The multivariate analysis results on IgG type and severe COVID-19 pneumonia development are shown in **Table 3**. Compared with the unvaccinated group, the OR value of the vac+IgG− group that developed into severe cases was 0.16 (0.03~0.82) without adjustment. Additionally, the OR value of the vac+IgG+ group that developed into severe cases was 0.04 (0.01~0.23). In model III, adjusted for all factors, the vac+IgG− group that developed into severe cases had an OR value of 0.21 (0.02–2.05), and the vac+IgG+ group had an OR value of 0.05 (0–0.63) compared with the unvaccinated group. In all four models, the OR values of the vac+IgG+ group were lower than those of the vac+IgG− and unvaccinated groups, which indicated that the protective efficacy of the vac+IgG+ group was stronger, and this trend was verified by a trend test (all OR values < 1).

**Table 3:** Multivariate analysis of the incidence of severe cases by vaccination combined with IgG level.

Variable	Non-adjusted model OR	Model I adj. OR	Model II adj. OR	Model III adj. OR
Unvaccinated	1 (ref)	1 (ref)	1 (ref)	1 (ref)
Vac+IgG−	0.16 (0.03~0.82)	0.19 (0.03~1.18)	0.17 (0.03~1.08)	0.21 (0.02~2.05)
Vac+IgG+	0.04 (0.01~0.23)	0.14 (0.02~0.87)	0.12 (0.02~0.75)	0.05 (0~0.63)
Trend test	0.2 (0.09~0.46)	0.35 (0.13~0.9)	0.32 (0.12~0.84)	0.22 (0.06~0.73)

Model I: adjusted for age + sex + BMI; model II: adjusted for model I + comorbidities; model III: adjusted for model II + albumin + IgM + platelets + lymphocytes + CRP.

### Basic information of severe cases

The basic information of the 10 cases that developed into severe cases is shown in **Table 4**, of whom only 2 were younger than 60 years and 8 were older than 60 years, 2 were women and 8 were men, and 2 had no underlying diseases and 8 had underlying diseases. Among them, 6 cases were in the unvaccinated group, 2 cases were in the vac+IgG− group, and 2 cases were in the vac+IgG+ group. **Table S3** shows that only 1 severity case had a booster vaccine injected. One case had a body mass index (BMI) of >30 kg/m^2^, and the remaining 9 cases had a BMI of 20.20–28.34 kg/m^2^. Patient 10, aged 31 years, had a BMI of 30.86 kg/m^2^. Patient 5, aged 41 years with a BMI of 20.70 kg/m^2^, was not vaccinated.

**Table 4:** Clinical characteristics of 10 severe cases.

No.	Sex	Age	BMI	Vaccine	IgG	IgM	LY	Fever	Cough	Dyspnea	Comorbidities
1	Male	67	23.44	No	0.01	0.03	0.77	Yes	Yes	Yes	Hypertension, heart disease
2	Female	71	25.71	No	0.01	0.23	1.00	Yes	No	No	Hypertension
3	Male	73	28.34	No	0.02	0.04	0.95	Yes	Yes	No	Hypertension, diabetes, heart disease
4	Male	72	25.90	No	0.03	0.03	1.21	No	No	No	Hypertension
5	Female	41	20.70	No	1.10	0.18	0.73	Yes	Yes	Yes	
6	Male	90	20.20	No	1.42	0.94	0.83	No	Yes	No	Hypertension
7	Male	72	24.69	Full	0.08	0.01	1.05	Yes	Yes	Yes	Heart disease
8	Male	76	23.88	Full	0.85	0.39	1.26	Yes	Yes	Yes	Heart disease
9	Male	68	27.34	Full	8.84	80.20	0.85	Yes	Yes	Yes	Hypertension, heart disease
10	Male	31	30.86	Booster	8.25	291.51	1.32	Yes	Yes	Yes	

## Discussion

Reports have suggested that vaccine efficacy against COVID-19 might have fallen since the Delta (B.1.617.2) severe acute respiratory syndrome coronavirus 2 (SARS-CoV-2) variant replaced the Alpha (B.1.1.7) variant as the predominant variant. The Delta variant had the highest risk of intensive care unit (ICU) admission and mortality (Lin et al., 2021). The efficacy of vaccines against severe COVID-19 pneumonia became the focus. In mRNA vaccine research, Lauring et al. found that compared with unvaccinated patients, COVID-19 severity rate was lower for vaccinated patients for each variant, including Alpha (OR: 0.33, 0.23–0.49), Delta (OR: 0.44, 0.37–0.51), and Omicron (OR: 0.61, 0.44–0.85) variants (Lauring et al., 2022). In China, inactivated vaccines were the main type. As of December 2021, the population has received 2,823.41 million doses of the COVID-19 vaccine. The research of Li et al. showed that while single-dose vaccination was not sufficiently protective, the two-dose dosing scheme of the inactivated vaccines was effective against the Delta variant infection in real-world settings (Li et al., 2021). The focus of this research was the relationship between vaccine dose and vaccine effectiveness. In our study, the level of IgG antibody production after vaccination and the protective effect on severe diseases were evaluated by classifying the vaccination status of the included patients, combined with the IgG results, and positive results were obtained.

Among the 580 cases, the severe case rate was 1.72% (10/580), among which the vaccinated patients were 0.77% (4/520) and the unvaccinated were 10.00% (6/60). It was in line with the incidence of severe cases in Guangzhou, China (Hu et al., 2021). This severe rate was lower than that of most hospitalized patients worldwide because China had hospitalized all COVID-19 cases, and the hospitalized population included more asymptomatic and mild patients, resulting in a lower severe rate of hospitalized patients (Lin et al., 2021; Lauring et al., 2022). The baseline data analysis revealed that children under 18 years of age had no severe cases, had milder symptoms, and had shorter hospital stays than adults. The incidence of severe cases in the elderly over 60 years old (10.96%) was higher than that in adults (0.39%), as shown in **Table S1**. Comorbidities were also one of the reasons for the high incidence of severe cases (7.84% for comorbidities and 0.42% for non-morbidities, **Table S3**). Morbidities were highly correlated with age. In this study, the morbidities of those over 60 years old (58.90%) were significantly higher than those in the other age groups (13.03% for 18–60 years old and 2.70% for less than 18 years old); thus, age was closely related to comorbidities and severe cases (**Table S1**). For the vaccination status, we performed no vaccine, partial (one dose), full (two doses), and booster (three doses) in the baseline analysis. Severe cases in the booster group were all lower than in the other three groups, and the length of hospital stay was shorter regardless of the presence or absence of underlying diseases, symptom incidence, CT lung lesions, supplemental oxygen, and ICU admission. This showed that the vaccine effectiveness of three vaccine doses was higher than two doses, one dose, and no vaccine, and three vaccine doses can make the body produce stronger vaccine efficacy, including the antibody levels. This was confirmed in the baseline data. The median IgG antibody level in the booster group was higher than that in the other groups, and the positive rate was 100% (50/50) (**Table 2**), which was also supported by related studies (Barda et al., 2021). Serological data showed that participants exhibit an initial enhancement in antibody levels by day 14, and the clearance of detectable SARS-CoV-2 protein correlated with the production of IgG (Gobbi et al., 2021; Ogata et al., 2022). The efficacy of mRNA vaccines against severe Delta declined in the first 10 weeks from the second dose but more slowly at 20 weeks (McKeigue et al., 2022). Vaccine efficacy against severe Delta did not diminish over time for patients who received two doses of the inactivated vaccine (Xia et al., 2022; Ye et al., 2022). The abovementioned studies showed that the protection of vaccination for severe cases was related to the antibody level. Some evidence suggested that vaccine protection against severe cases does not diminish over time, but this may be related to the short duration between vaccination and the onset of disease in the study. Our study focused more on the relationship between IgG levels after vaccination and protection in severe cases. The univariate analysis of risk factors related to severe cases in this study showed that the incidence of critical illness in the vac+IgG− group was 0.84 times lower than that in the unvaccinated group, which seems to indicate that vaccine still had a certain protective effect against severe cases although IgG antibodies were not detected after vaccination. However, the vac+IgG− group had the longest transformation time and the lowest level of inflammatory indexes, which may be related to the low immune response of the body. This group of patients deserves more attention from clinicians.

The univariate analysis (**Figure 1**) revealed many factors related to the occurrence of severe cases. In addition to laboratory indicators, age and comorbidities cannot be ignored, which were also found in our supplementary data. Older age (>60 years) and patients with comorbidities were more likely to develop into severe cases. Considering the influence of many factors, the multivariate analysis had three models according to different covariates. The results of the three models all showed that when age, BMI, comorbidities, and laboratory tests were considered, no statistically significant difference was found between the vac+IgG− group and the unvaccinated group in the occurrence of severe cases. The vac+IgG+ group had a more protective effect on the occurrence of severe cases and showed a better protective effect after adjusting for different confounding factors. This proved that doctors should pay attention to the possibility of developing severe cases in elderly patients and with comorbidities, who had not been vaccinated or IgG− after vaccination, in the process of clinical diagnosis and treatment.

The clinical characteristics of all severe cases were presented in this study (**Table 4**), of whom most were elderly, with comorbidities, and were unvaccinated. Two special severe cases, aged 31 and 41 years, were IgG-positive patients without comorbidities. The 31-year-old male patient was injected with a booster, and the level of IgM reached 291.51 S/CO, but his BMI was 30.86 kg/m^2^. The intense immune response and high BMI resulted in overburdened lungs and increased disease severity. Research suggested that obesity was linked to COVID-19 severity (Hussain et al., 2020; Michalakis and Ilias, 2020; Zhou et al., 2021). The 41-year-old female patient, who was not vaccinated, had persistent pulmonary exacerbations as the disease progressed and was on respiratory support; although we cannot exactly know how this patient developed into a severe case, the patient was not effectively protected by the vaccine.

This study had limitations. Firstly, there were a few severe cases, which may not satisfy the multivariate analysis. However, we analyzed the results from multiple perspectives to minimize the bias of the study. Secondly, vaccination was roughly divided into vaccinated and unvaccinated, and the relationship between the number of vaccinations combined with IgG levels and the occurrence of critical illness was not analyzed because the subgroup analysis could not be done due to the limited number of outcome events. However, we demonstrated from the supplementary data that our results should be plausible.

According to the data analysis, effective vaccination (producing sufficient levels of IgG antibodies) can reduce the risk of COVID-19 that progresses to severe cases, while three doses of the inactivated vaccine can significantly increase the IgG-positive rate in the population. The elderly and people with comorbidities who have not been vaccinated and who have not produced IgG antibodies after vaccination should pay close attention to the possibility of developing severe cases and should be intervened in advance if necessary.

## Data availability statement

The original contributions presented in the study are included in the article/**Supplementary Material**. Further inquiries can be directed to the corresponding author.

## Ethics statement

Written informed consent was obtained from the individual(s) for the publication of any potentially identifiable images or data included in this article.

## Author contributions

HY, JM, FS, and YX conceived and designed the experiments. AL, JL, HL, YChen, and YY performed the experiments. YCheng, BH, and YX analyzed and interpreted the results. HY, JM, and AL wrote the manuscript. All authors contributed to the article and approved the submitted version.

## Funding

This work was supported by the Key Research and Development Program of Shaanxi Province (grant number: 2020SF-109).

## Acknowledgments

We thank YC and BH (School of Public Health, Xi’an Jiaotong University) for their help and guidance in the statistical analysis of the data.

## Conflict of interest

The authors declare that the research was conducted in the absence of any commercial or financial relationships that could be construed as a potential conflict of interest.

The reviewer TZ declared a shared affiliation, with no collaboration, with several of the authors, YC, BH, and HL, to the handling editor at the time of review.

## Publisher’s note

All claims expressed in this article are solely those of the authors and do not necessarily represent those of their affiliated organizations, or those of the publisher, the editors and the reviewers. Any product that may be evaluated in this article, or claim that may be made by its manufacturer, is not guaranteed or endorsed by the publisher.
